# Risk factors for the accuracy of the initial diagnosis of malaria cases in China: a decision-tree modelling approach

**DOI:** 10.1186/s12936-021-04006-4

**Published:** 2022-01-07

**Authors:** Gang Li, Donglan Zhang, Zhuo Chen, Da Feng, Xinyan Cai, Xiaoyu Chen, Shangfeng Tang, Zhanchun Feng

**Affiliations:** 1 School of Medicine and Health Management, Tongji Medical College, Huazhong University of Science and Technology, 430030 Wuhan, Hubei China; 2 Department of Health Policy and Management, College of Public Health, University of Georgia, Athens, GA 30602 USA; 3 School of Economics, University of Nottingham Ningbo China, Ningbo, 531200 Zhejiang China; 4 School of Pharmacy, Tongji Medical College, Huazhong University of Science and Technology, Wuhan, 430030 Hubei China; 5 Department of Epidemiology and Biostatistics, University of Georgia, Athens, GA 30602 USA

**Keywords:** Geographic variation, Health seeking behaviour, Healthcare institutions, Decision tree

## Abstract

**Background:**

Early accurate diagnosis and risk assessment for malaria are crucial for improving patients’ terminal prognosis and preventing them from progressing to a severe or critical stage. This study aims to describe the accuracy of the initial diagnosis of malaria cases with different characteristics and the factors that affect the accuracy in the context of the agenda for a world free of malaria.

**Methods:**

A retrospective study was conducted on 494 patients admitted to hospitals with a diagnosis of malaria from January 2014 through December 2016. Descriptive statistics were calculated, and decision tree analysis was performed to predict the probability of patients who may be misdiagnosed.

**Results:**

Of the 494 patients included in this study, the proportions of patients seeking care in county-level, prefecture-level and provincial-level hospitals were 27.5% (n = 136), 26.3% (n = 130) and 8.3% (n = 41), respectively; the proportions of patients seeking care in clinic, township health centre and Centres for Disease Control and Prevention were 25.9% (n = 128), 4.1% (n = 20), and 7.9% (n = 39), respectively. Nearly 60% of malaria patients were misdiagnosed on their first visit, and 18.8% had complications. The median time from onset to the first visit was 2 days (IQR: 0-3 days), and the median time from the first visit to diagnosis was 3 days (IQR: 0–4 days). The decision tree classification of malaria patients being misdiagnosed consisted of six categorical variables: healthcare facilities for the initial diagnosis, time interval between onset and initial diagnosis, region, residence type, insurance status, and age.

**Conclusions:**

Insufficient diagnostic capacity of healthcare facilities with lower administrative levels for the first visit was the most important risk factor in misdiagnosing patients. To reduce diagnostic errors, clinicians, government decision-makers and communities should consider strengthening the primary care facilities, the time interval between onset and initial diagnosis, residence type, and health insurance status.

## Background

Malaria is a major infectious disease that continues to present challenges to population health and healthcare systems worldwide, particularly in developing countries [1, 2], where it is a major cause of mortality and morbidity [1, 3]. According to the latest data, an estimated 229 million malaria cases and 409,000 deaths occurred globally in 2019 [3]. China was once a major malaria-endemic country, but the Chinese Government has made great strides in preventing and controlling malaria [4]. After 70 years, there has been no report of autochthonous malaria cases for nearly four consecutive years, since 2017 [5, 6], meeting the goal of malaria elimination set by the World Health Organization [7, 8].

With the sharp increase in international travel among Chinese people, the risk of imported malaria cases from malaria-endemic areas threatens the maintenance of the malaria elimination goal of China [9]. Among imported malaria cases, *Plasmodium falciparum* is the most common species, with potentially fatal outcomes [10]. The fatality of malaria cases in China is rising, with more than four-fifths of deaths associated with severe complications, such as severe brain/liver/kidney lesions, shock, and haemolysis [11, 12].

It is recommended that all malaria cases be treated effectively and affordably within 24 h of onset [13]. However, not all patients can get a timely and accurate diagnosis. Most cases of “malaria” (i.e., having a fever) are self-diagnosed, and most treatments and deaths occur at home [14]. Early and accurate diagnosis and risk assessment for malaria is crucial for improving patients’ terminal prognosis and preventing them from progressing to the severe or critical state [15]. In addition, it also helps to reduce the direct or indirect treatment costs for patients and the risk of community transmission [16].

To date, studies identified some factors associated with the misdiagnosis of malaria, including insufficient diagnostic equipment, means of diagnosis, lack of clinical supervision/training for local clinicians, lack of malaria-related knowledge for travellers, *Plasmodium* species, and health system factors [17–24]. However, most of these have been carried out in a few malaria-endemic developing countries or in just one province of China [25]. Studies on misdiagnosis of malaria using samples from multiple provinces and rich clinical medical records in China are limited. This study aims to identify factors associated with the misdiagnosis of malaria and develop a predictive model based on the medical records to guide prevention and reduce misdiagnoses.

## Methods

### Study design and site

A multi-stage sampling approach was adopted to obtain the study population. In each region of China, two provinces with the highest incidence of malaria were selected, including Zhejiang and Jiangsu in Eastern China, Henan and Anhui in Central China, and Yunnan and Sichuan in Western China [26]. A preliminary analysis was conducted on the number of malaria cases reported by different levels of hospitals based on the data provided by the National Health Commission. It was found that most county-level hospitals reported fewer than two cases, and most cases were reported in the higher level of hospitals. Then two provincial-level hospitals, five prefecture-level hospitals, 10 county-level hospitals with relatively large numbers of malaria cases in each province were selected according to the hospitals’ lists of reported malaria cases during 2014-2016, and the cluster sampling method was used to collected patients’ medical records in each selected hospital. According to the sampling design, a total of 1,868 cases from 102 hospitals should be investigated. In fact, 1,633 cases were collected from 63 hospitals because some hospitals surveyed refused to provide medical records. After screening the completeness of patients’ medical records reported by each hospital, 494 cases from 26 hospitals were finally included in this analysis.

**Fig. 1:**
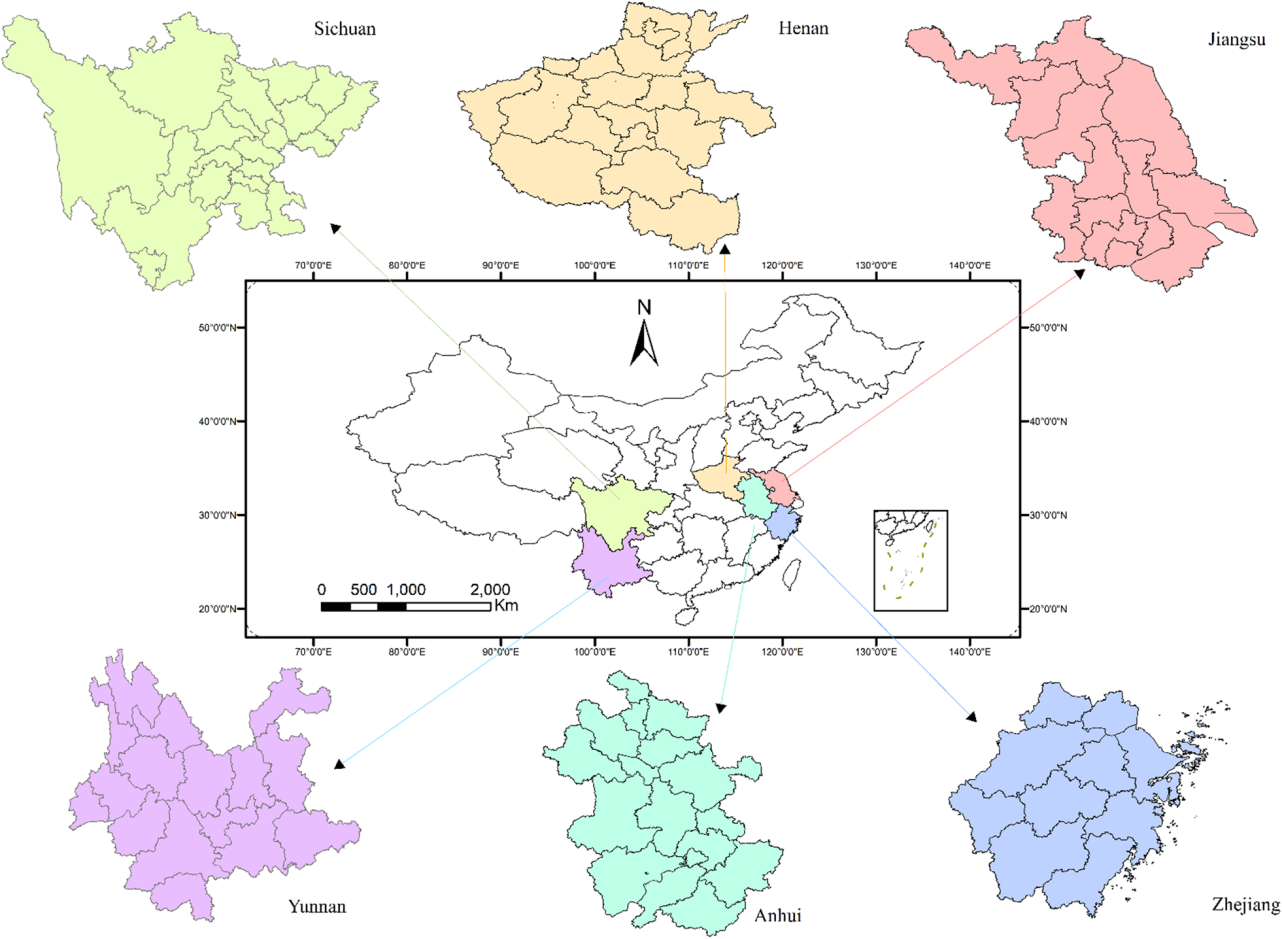
Geographic distribution of the six selected provinces in China

Figure 1 shows the geographic distribution of the six selected provinces. Jiangsu and Zhejiang provinces are geographically in proximity to the East China Sea, considered one of the most developed regions in the country and has frequent trade exchanges with other countries. Henan and Anhui are China’s inland provinces, bordering each other, with flat terrain. High-speed rail and highways pass through these two provinces connecting with most other provinces in the country. Sichuan and Yunnan are mountainous and relatively underdeveloped areas located in Southwestern China. Transport infrastructure and medical facilities are relatively scarce in these two provinces [27].

### Data collection

This is a retrospective study of 494 patients admitted to hospitals with a diagnosis of malaria from January 2014 through December 2016. These patients were confirmed to be positive for *Plasmodium* species by rapid diagnostic tests (RDTs), microscopy or polymerase chain reaction (PCR). Data were obtained from the patients’ medical records using a protocol designed for the study. Demographic, epidemiological and clinical data were collected from medical records, including age, gender, occupation, education, residence type, health insurance status, detailed course from the onset of symptoms to seeking medical services, treatment details, co-morbidities, outcome (cure or death), as well as blood chemicals and microbiology analyses.

### Definitions

China has five levels of administrative divisions: provincial, prefectural, county, township, and village. There are two main types of health facilities in China [28]. One is a professional public health agency, such as various levels of Centre for Disease Control and Prevention (CDC), which undertake surveillance and detection of infectious disease, prevention of chronic disease and distribution of certain free medications, including anti-malarial drugs. The other type of facility are institutions that provide treatment services, including hospitals, township health centres and village clinics. CDCs and hospitals can be divided into three levels: provincial, prefectural and county level, aligned with the administrative levels. Township health centres and clinics are generally set up within the jurisdiction of townships and villages. In most cases, medical facilities with higher administrative levels also have higher diagnostic and treatment capabilities.

### Statistical analysis

The descriptive statistics, Chi-square and t-test analyses were performed with Stata version 15.0 (StataCorp, College Station, TX, USA). Descriptive statistics were used to summarize the sociodemographic characteristics. Statistical significance was defined as a p-value < 0.05. Decision tree analysis using the IBM SPSS Statistics 25.0 (IBM Corp, Armonk, NY, USA) was performed to build an optimum and significant predictive model to predict the probabilities of misdiagnosing patients. The classification and regression tree (CART) techniques were conducted to obtain the best cut-off points in the software [29, 30]. The initial diagnosis (misdiagnosis/correct diagnosis) was treated as a dependent variable, and demographic and clinical characteristics variables (age, gender, marital status, occupation, residence type, region, insurance status, *Plasmodium* species, time interval between onset and initial diagnosis, healthcare facilities for initial diagnosis) were treated as as independent variables in the model. Parameters of the final CART model were set as follows: maximum tree depth was five, with minimum cases in parent node as 10 and in child node as five.

### Ethical approval and consent

The study was approved by the Ethics Committee of the Tongji Medical College of Huazhong University of Science and Technology (IORG0003571). Written permission to access and analyse the research data was granted by the National Health Commission of the People’s Republic of China and the administrators of each hospital. Patient information was anonymized and de-identified.

## Results

### Sociodemographic characteristics

The demographic and clinical characteristics of patients are summarized in Table 1. The median age of the 494 patients was 39 years (range: 11-63 years), and most were male (98.2%). Of these individuals, around 80% were married, 62% lived in rural areas, 35% were employed in agriculture, and 70% had health insurance. The malaria cases were mostly from *Plasmodium falciparum*: 62.4% (n = 308), and cases due to *Plasmodium vivax*, *Plasmodium ovale*, and *Plasmodium malariae* were 19.2% (n = 95), 3.0% (n = 15), and 0.6% (n = 3), respectively.

**Table 1:** Epidemiological characteristics of individuals with imported malaria

Factors	Number (%)
Gender (n = 494)	
Male	485 (98.2%)
Female	9 (1.8%)
Age (years) (n = 494)	
< 35	205 (41.5%)
≥ 35	289 (58.5%)
Marital Status (n = 494)	
Married	394 (79.8%)
Single/divorce/separated	100 (20.2%)
Occupation (n = 494)	
Agriculture	170 (34.4%)
No-agriculture	324 (65.6%)
Residence (n = 489)	
Rural	304 (62.2%)
Urban	185 (37.8%)
Region (n = 494)	
Eastern	67 (13.6%)
Central	309 (62.6%)
Western	118 (23.9%)
Plasmodium species (n = 494)	
* Unclassified*	68 (13.8%)
* Plasmodium falciparum*	308 (62.4%)
* Plasmodium ovale*	15 (3.0%)
* Plasmodium vivax*	95 (19.2%)
* Plasmodium malariae*	3 (0.6%)
* Mixed*	5 (1.0%)
Rank of healthcare facilities for the initial diagnosis (n = 494)	
Village clinic	128 (25.9%)
Township health center	20 (4.1%)
County-level CDC	22 (4.5%)
Prefecture-level CDC	15 (3.0%)
Provincial-level CDC	2 (0.4%)
County-level hospital	136 (27.5%)
Prefecture-level hospital	130 (26.3%)
Provincial-level hospital	41 (8.3%)
Initial diagnosis (n = 461)	
Misdiagnosis	265 (57.5%)
Correct diagnosis	196 (42.5%)
Admission pathway (n = 494)	
Emergency	244 (49.4%)
Outpatient	242 (49.0%)
Referral	8 (1.6%)
Symptom (n = 494)	
Fever	491 (99.4%)
Chills	209 (42.3%)
Sweating	191 (38.7%)
Splenomegaly	7 (1.4%)
Anemia	41 (8.3%)
Periodic	123 (24.9%)
Complications developed (n = 494)	
Yes	93 (18.8%)
No	401 (81.2%)
Insurance status (n = 494)	
Insured	346 (70.0%)
Uninsured	148 (30.0%)
Hospitalization cost (USD) (n = 486)	
36–500	106 (21.8%)
501–1000	136 (28.0%)
1001–2000	161 (33.1%)
2001–34630	83 (17.1%)
Days	Mean, Median, Min, P10, P25, P75, P90, Max
Time interval from fever onset to initial diagnosis	2.0, 1, 0, 0, 3, 5,20
Time interval from initial diagnosis to final diagnosis	3.5, 2, 0, 0, 4, 8, 90
Time interval from fever onset to final diagnosis	5.5, 4, 1, 2, 7, 10, 90

## Health-seeking behaviour

The proportions of patients seeking care in county-level, prefecture-level and provincial-level hospital were 27.5% (n = 136), 26.3% (n = 130) and 8.3% (n = 41), respectively; the proportions of patients seeking care in clinic, township health centre, and CDC were 25.9% (n = 128), 4.1% (n = 20) and 7.9% (n = 39), respectively. Nearly 60% of malaria patients were misdiagnosed on their first visit, and concerning complications affected 18.8% of the patients. Approximately half of the patients were admitted to the hospital via emergency rooms. Patients with fever, chills and sweating upon admission were 99.4% (n = 491), 42.3% (n = 209), and 38.7% (n = 191), respectively.

Median time intervals were as follows: from onset to the first medical visit, 2 days (IQR: 0–3 days); from the first medical visit to diagnosis, 3 days (IQR: 0–4 days); and, from onset to final diagnosis, 5.5 days (IQR: 2–10 days). Table 2 shows the time interval from symptom onset to initial diagnosis, and from initial diagnosis to final diagnosis of malaria cases in multiple healthcare institutions. The time interval between onset and initial diagnosis in the provincial-level, prefecture-level and county-level hospitals was 3.3 days, 2.4 days and 2.2 days, respectively, which was longer than in other healthcare institutions. The time interval from the initial diagnosis to the final diagnosis in prefecture-level, provincial-level and county-level CDCs was 0.5 days, 1.0 days and 1.1 days, respectively, followed by provincial-level hospitals (1.1 days) and prefecture-level hospitals (1.3 days). However, the time interval from the initial diagnosis to the final diagnosis in village clinics, county-level hospitals and township health centres was much longer at 5.8 days, 4.8 days and 4.0 days, respectively.

**Table 2:** Time interval from symptom onset to initial diagnosis, and from initial diagnosis to final diagnosis of malaria cases in multiple healthcare institutions

Healthcare institutions	Time interval between onset and initial diagnosis Mean (95% CI)	Time interval from initial diagnosis to final diagnosis Mean (95% CI)
Village clinic	1.42 (0.92–1.91)	5.80 (4.42–7.17)
Township health center	1.05 (0.28–1.82)	4.00 (2.43–5.57)
County-level CDC	1.32 (0.59–2.05)	1.09 (0.11–2.07)
Prefecture-level CDC	1.77 (0.85–2.68)	0.47 (0.09–0.84)
Provincial-level CDC	1.25 (− 0.22 to 2.72)	1.00 (− 0.96 to 2.96)
County-level hospital	2.21 (1.78–2.63)	4.83 (3.28–6.38)
Prefecture-level hospital	2.40 (2.00–2.80)	1.29 (0.82–1.77)
Provincial-level hospital	3.32 (2.09–4.55)	1.12 (0.44–1.81)

## Factors influencing initial diagnosis and malaria complications

Demographic and epidemiological factors influencing initial diagnosis are shown in Table 3. There was a significant difference in misdiagnosis rate for the initial diagnosis between different regions, *Plasmodium* species and healthcare facilities. Among patients from Western China, around 67.5% (n = 79) of patients were not correctly diagnosed; misdiagnosis rates in Central and Eastern China were 58.8% (n = 163) and 34.3% (n = 23), respectively. Among all mixed malaria cases, 80.0% (n = 4) were not correctly diagnosed at the first medical visit. The misdiagnosis rates of mixed, unclassified, *P. ovale*, *P. falciparum*, *P. vivax*, and *P. malariae* were 80.0, 71.7, 68.5, 51.4, 46.7, and 33.3%, respectively. Notably, the misdiagnosis rate was up to 100% at clinics and township health centres, and 68.5% in county-level hospitals during their first medical visit. More generally, the rate of misdiagnosis decreased with the level and quality of health institutions.

**Table 3:** Influential factors of the malaria cases at initial diagnosis

Influential factors	Initial diagnosis (% or 95% CI)		P-value
	Misdiagnosis(%)	Correct diagnosis(%)	
Gender			
Male	259 (57.3)	193 (42.7)	0.574
Female	6 (66.7)	3 (33.3)	
Age (years)	38.4 (37.2–39.5)	38.3 (37.0–39.6)	0.930
Marital Status			
Married	217 (58.8)	152 (41.2)	0.250
Single/divorce/separated	48 (52.2)	44 (47.8)	
Occupation			
Agriculture	94 (59.1)	65 (40.9)	0.606
No-agriculture	171 (56.6)	131 (43.4)	
Residence			
Rural	161 (57.1)	121 (42.9)	0.841
Unban	101 (58.1)	73 (42.0)	
Region			
Eastern	23 (34.3)	44 (65.7)	0.000
Central	163 (58.8)	114 (41.2)	
Western	79 (67.5)	38 (32.5)	
Insurance status			
Insured	188 (58.0)	136 (42.0)	0.718
Uninsured	77 (56.2)	60 (43.8)	
Plasmodium species			
* Unclassified*	43 (71.7)	17 (28.3)	0.006
* Plasmodium falciparum*	147 (51.4)	139 (48.6)	
* Plasmodium ovale*	63 (68.5)	29 (31.5)	
* Plasmodium vivax*	7 (46.7)	8 (53.3)	
* Plasmodium malariae*	1 (33.3)	2 (66.7)	
* Mixed*	4 (80.0)	1 (20.0)	
Rank of healthcare facilities for the initial diagnosis			
Village clinic	112 (100.0)	0 (0.0)	0.000
Township health center	18 (100.0)	0 (0.0)	
County-level CDC	6 (30.0)	14 (70.0)	
Prefecture-level CDC	6 (40.0)	9 (60.0)	
Provincial-level CDC	1 (50.0)	1 (50.0)	
County-level hospital	87 (68.5)	40 (31.5)	
Prefecture-level hospital	27 (21.3)	100 (78.7)	
Provincial-level hospital	8 (20.0)	32 (80.0)	
Time interval between onset and initial diagnosis	1.6 (1.3–1.9)	2.7 (2.3–3.1)	0.000

No patients infected with *P. malariae* and mixed species developed complications. Nearly 20% of patients infected with *P. falciparum* had complications. Except for prefecture-level hospitals, the incidence of complications was relatively low for patients whose first visit was at a higher administrative level of healthcare institution. There were 0, 4.9 and 8.1% complications in patients initially visiting provincial-level CDCs, provincial-level hospitals and county-level hospitals, respectively. In contrast, there were 28.1, 26.9 and 20.0% complications in patients initially visiting village clinics, prefecture-level hospitals and township health centres, respectively. The proportion of complications in patients with a correct initial diagnosis was 13.3%, whereas, it was 23.3% in patients with an incorrect initial diagnosis. Furthermore, malaria patients with complications tended to have a significantly longer time interval between onset and initial diagnosis (Table 4).

**Table 4:** Influential factors for complications developed among the malaria cases

Influential factors	Complications developed (% or 95% CI)		P value
	Yes	No	
Gender			
Male	91 (18.8)	394 (81.2)	0.793
Female	2 (22.2)	7 (77.8)	
Age (years)	38.8 (36.9–40.8)	38.2 (37.3–39.2)	
Marital Status			
Married	71 (18.0)	323 (82.0)	0.363
Single/divorce/separated	22 (22.0)	78 (78.0)	
Occupation			
Agriculture	23 (13.5)	147 (86.5)	0.029
No-agriculture	70 (21.6)	254 (78.4)	
Residence			
Unban	55 (18.1)	249 (81.9)	0.503
Rural	38 (20.5)	147 (79.5)	
Region			
Eastern	9 (13.4)	58 (86.6)	0.000
Central	84 (27.2)	225 (72.8)	
Western	0 (0.0)	118 (100.0)	
Insurance status			
Insured	68 (19.7)	278 (80.4)	0.472
Uninsured	25 (16.9)	123 (83.1)	
Plasmodium species			
* Unclassified*	24 (35.3)	44 (64.7)	0.000
* Plasmodium falciparum*	61 (19.8)	247 (80.2)	
* Plasmodium ovale*	6 (6.3)	89 (93.7)	
* Plasmodium vivax*	2 (13.3)	13 (86.7)	
* Plasmodium malariae*	0 (0.0)	3 (100.0)	
* Mixed*	0 (0.0)	5 (100.0)	
Rank of healthcare facilities for the initial diagnosis			
Village clinic	36 (28.1)	92 (71.9)	0.000
Township health center	4 (20.0)	16 (80.0)	
County-level CDC	3 (13.6)	19 (86.4)	
Prefecture-level CDC	2 (13.3)	13 (86.7)	
Provincial-level CDC	0 (0.0)	2 (100.0)	
County-level hospital	11 (8.1)	125 (91.9)	
Prefecture-level hospital	35 (26.9)	95 (73.1)	
Provincial-level hospital	2 (4.9)	39 (95.1)	
Initial diagnosis			
Misdiagnosis	61 (23.0)	204 (77.0)	0.008
Correct diagnosis	26 (13.3)	170 (86.7)	
Time interval between onset and initial diagnosis	1.4 (0.9–2.0)	2.1 (1.8–2.4)	0.030
Time interval between initial diagnosis and final diagnosis	4.2 (3.1–5.4)	3.1 (2.5–3.7)	0.080
Time interval between onset and final diagnosis	5.7 (4.5–6.9)	5.3 (4.7–6.0)	0.631

## Decision tree classifier model

**Fig. 2:**
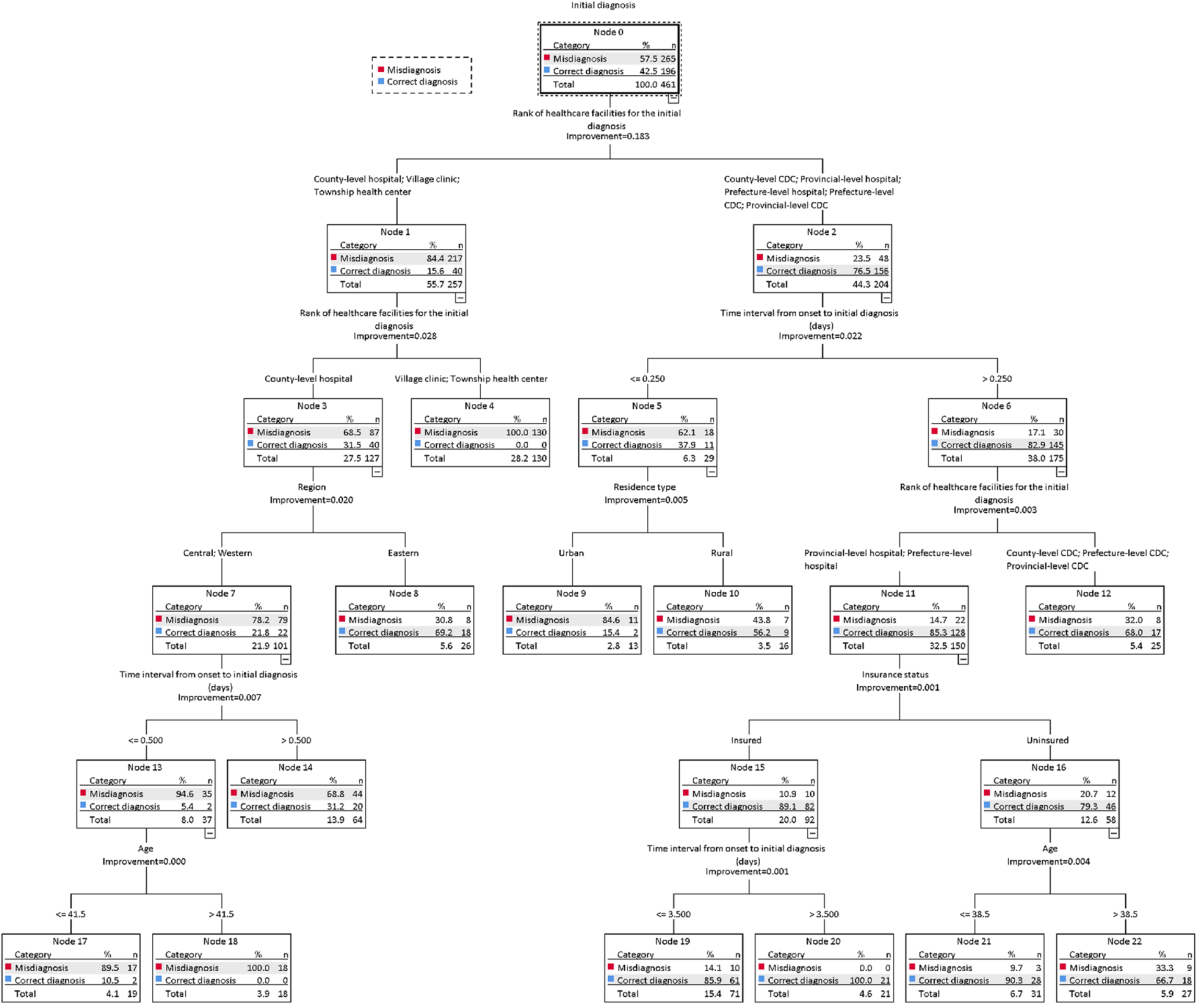
Tree model of initial diagnosis as dependent variable by CART method

Figure 2 shows the decision tree classification of malaria patients being misdiagnosed. This model was useful to identify the risk factors with the introduction of sub-groups with similar risk levels and the risk factors for initial misdiagnosis in the studied patients. The decision tree classification of malaria patients being misdiagnosed consisted of six categorical variables that ranked by their importance: level of healthcare facilities for the initial diagnosis, time interval between onset and initial diagnosis, region, residence type, insurance status, and age. The accuracy of the prediction model was 85.0%.

The decision tree shows that in a sub-group of patients who visited clinics and township health centres for the first time, the probability of a patient being misdiagnosed was 100%. In the sub-group of patients who visited county-level hospitals for the first time, with patients from Eastern China, the probability of being misdiagnosed was 30.8%. In the same situation with patients from Central and Western China, if the time interval between onset and initial diagnosis >0.5 days, the probability of being misdiagnosed was 68.4%, while if the time interval between onset and initial diagnosis ≤0.5 days and age >41.5 years, there was a 100% probability of being misdiagnosed. In the sub-group of patients who visited healthcare facilities at county-level and above for the first time, whose time interval between onset and initial diagnosis was <0.25 days, and who lived in urban areas, 84.6% of individuals was misdiagnosed. In the same situation with the time interval between onset and initial diagnosis >0.25 days, if the patients sought care at different levels of CDC, 32.0% of individuals were misdiagnosed.

## Discussion

This study described the accuracy of the initial diagnosis of malaria cases with different characteristics, as well as the factors that affect the accuracy. This study also analysed the importance of misdiagnosis influencing factors through the CART model. It was found that the level of healthcare facility for the initial visit was the most important factor affecting whether malaria patients were correctly diagnosed. The proportion of misdiagnosed patients seeking care in township health centres and clinics was 100%. The proportion of misdiagnosed patients seeking care in county-level hospitals dropped to about 70%. Even for patients seeking care at different levels of CDC in China, the rate of misdiagnosis was still very high, which was far from the goal of the National Malaria Elimination Programme (NMEP) [31].

In 2010, the Chinese Government launched the NMEP (2010-2020) to enable malaria patients to receive timely treatment and reduce delays. Through this action plan, the government provided a large amount of funding to health facilities at all levels to train clinicians to perform blood tests for malaria parasites [32]. The survey year (2014-2016) was very close to 2020, and the goal of this year 2020 was to equip health workers at all levels of facilities with the capacity to diagnose and treat malaria patients. However, according to this investigation, it was observed that even though the National Health Commission had rolled out RDTs to all levels of health institutions, neither public health institutions, such as CDCs nor hospitals could accurately diagnose malaria. With the elimination of local malaria, clinicians at all levels of health institutions, especially those at or below county level, have not been exposed to malaria cases for a long time, which could have easily led clinicians to inaccurately diagnose malaria patients with fever as having common colds and neglecting to ask about their travel history [33, 34]. Moreover, it is also possible that these institutions may neither have the facilities required for diagnosis nor have physicians qualified to diagnose malaria [35]. Most cases were imported falciparum malaria in the years studied, particularly in regions bordering countries in Southeast Asia [36]; the patients were usually in serious condition [37, 38]. Clinicians in health institutions at or below county level may use their experience to make a preliminary malaria diagnosis. However, accurate diagnosis requires microscopy tests and PCR. They would recommend patients to a higher-level hospital or CDC for further diagnosis and treatment.

It is noted that the time interval from the initial diagnosis to final diagnosis for patients seeking care initially in county-level hospitals, township health centres and clinics was longer than that of patients seeking care in higher-level hospitals or CDCs. If a patient is diagnosed with malaria in time, clinicians could develop an appropriate treatment plan, which would significantly reduce the occurrence of complications and prevent the progression of the disease [3]. At the same time, it could reduce the cost of treatment and the economic burden on patients. The disparity in the time interval from initial diagnosis to final diagnosis for patients seeking care between different levels of health facilities should be noted. Patients living near higher-level hospitals usually have better access to timely and high-quality medical services than patients living in rural areas. In particular, diseases such as malaria are easily misdiagnosed as common colds [39]. If primary health institutions cannot make a correct diagnosis for patients from rural areas, it will greatly increase the probability of their condition worsening. After achieving the goal of eliminating malaria, it is necessary to build sustainable capacity of health workers and facilities at different levels to diagnose malaria, and establish a rapid referral system between primary care institutions and higher-level hospitals designated for malaria treatment in each prefecture-level area.

In this study, it was found that misdiagnosis rates between different regions varied greatly. Patients living in Western China were more likely to be misdiagnosed than those living in Eastern and Western China. The disparity of patients seeking care in different regions should be noted. Rural patients living in Western China, due to the inconvenience of transportation, usually sought care in nearby clinics and township health centres [25]. Due to insufficient diagnosis and treatment capabilities of local clinicians [21, 40], it was easy to cause patients to be misdiagnosed and delayed. There is a need to: allocate more anti-malarial funds to the western regions bordering countries in Southeast Asia; train qualified doctors in RDT techniques; and, educate communities and households in order to close the health gap between regions. The risk of resurgence of local transmission from imported malaria is not only a public health threat in China, but also in countries where malaria has been eliminated [41]. To maintain malaria-free status in regions where malaria transmission has been interrupted, cross-border collaborations with countries in the region will be beneficial. To consolidate progress in malaria eradication, it is necessary for China to strengthen its cooperation with Southeast Asia and African countries in malaria control and research [42].

## Limitations of the study

This study has several limitations. First, some variables used in the analysis need to be extracted from the medical records. A limited number of hospitals surveyed can provide complete medical records. Patients with incomplete electronic medical records were excluded from this study. Second, some hospitals surveyed refused to provide medical records, thus the actual collected cases are smaller than expected. Third, as to whether a patient was correctly diagnosed, some clinicians may not rely on gold standard RDTs and microscopic examination but rely on personal experience to diagnose that the patient was infected with *Plasmodium* parasites and then referred the patient to a higher-level institution for further diagnosis. If, however, the clinician was unable to make a definite diagnosis, it was considered a misdiagnosis. The consequence is that misdiagnosis may be overestimated. Fourth, some variables, such as literacy level and self-care at home by patients may be associated with the timeliness of seeking care. However, due to the limitation of utilizing medical records, which did not provide such variables, the possible confounding effect of these variables were not explored. Finally, malaria cases date from 2014 to 2016, which were collected five years ago, and may not reflect the more recent situation.

## Conclusions

Insufficient diagnostic capacity of healthcare facilities at lower administrative levels for the initial visit was the most important risk factor in diagnosing malaria patients. This study suggests that clinicians, government decision-makers, communities, and employers should consider comprehensively the healthcare facilities for an initial diagnosis, the time interval between onset and initial diagnosis, residence type, and health insurance status in order to reduce the misdiagnosis rate.

## Data Availability

The datasets used in the current study are available from the corresponding author and will also be presented as a supplemental file to the article.

## Abbreviations

WHO: World Health Organization
NMEP: National Malaria Elimination Programme
RDTs: Rapid Diagnostic Tests
CDCs: Centers for Disease Control and Prevention
